# Implantation of a Small Aperture Intraocular Lens in Eyes with Irregular Corneas and Higher Order Aberrations

**DOI:** 10.18502/jovr.v17i3.11568

**Published:** 2022-08-15

**Authors:** Fabrizio Franco, Marco Branchetti, Lidia Vicchio, Federica Serino, Marco Piergentili, Vito Spagnuolo, Francesca Santoro, Gianni Virgili, Fabrizio Giansanti

**Affiliations:** ^1^Department of Ophthalmology, Careggi Teaching Hospital, Florence, Italy; ^2^Department of Neurosciences, Faculty of Psychology, University of Florence, Pharmacology and Children Health, Florence, Italy

**Keywords:** Aberrations, Cataract, IC-8, Pinhole

## Abstract

**Purpose:**

Corneal irregularities can lead to high order aberrations (HOAs) and may influence the outcomes in terms of intraocular lens (IOL) selection and visual acuity assessment. The aim of this study was to evaluate the visual acuity and satisfaction after IC-8 implants in patients characterized by corneal irregularities and HOAs who could not undergo refractive surgery due to the poor residual thickness of the cornea or other conditions such as astigmatism secondary to previous radial keratotomy.

**Methods:**

This descriptive, retrospective cohort study was conducted on nine eyes in six patients affected by corneal irregularities and HOAs who had undergone IC-8 IOL implantation. The primary endpoint was the best-corrected visual acuity (BCVA), the subjective visual function, and the visual field.

**Results:**

Nine eyes of six patients (three bilateral implantation) were enrolled. For each patient, BCVA, vision, and lifestyle quality were evaluated. In all patients, we noticed an improvement in all parameters without visual field defects.

**Conclusion:**

Our work encourages the use of the IC8 lens to improve visual acuity in patients with irregular corneas and HOAs who cannot be treated with customized refractive surgery. Patients experience a subjective improvement of their quality of vision and also more self-confidence in their daily life. IC-8 lenses do not interfere with the visualization of retinal fundus and there is no impairment of the visual field detected by patients.

##  INTRODUCTION

Cataract surgery is the most performed ophthalmic procedure worldwide. Patients have higher expectations regarding postoperative refractive results and relative independence from spectacles, than in the past.^[1]^

To determine the desired refractive results, a complete biometric measure should be performed to calculate the power of the intraocular lens (IOL). Central corneal power (K) is an important factor in the calculation formula to decide the power of the IOL to be implanted.^[2]^

Nowadays, Scheimpflug and OCT-swept source ray tracing technology allow to study in a better way the anterior and posterior faces of the cornea with the aim of estimating its total power.^[3]^ Corneal irregularities could lead to high order aberrations (HOAs) that can influence the outcomes in terms of IOL selection and visual acuity.^[4]^

Recently, AcuFocus (Irivne, CA) has made available IC-8: a one-piece, acrylic, hydrophobic, posterior chamber small-aperture IOL that provides an increase in the range of vision from far to near, by extending the depth of focus.^[5]^

It works by using the principle of the pinhole to improve visual acuity and minimize dysphotopsia symptoms by applying the same small-aperture principle optics as the Kamra inlay: where peripheral rays are stopped while central light rays are allowed to pass with no interference and focus on the retina.^[6]^

It is a biconvex lens with a total length of 12.50 mm and a diameter of 6 mm composed of polyvinylidene fluoride and nanoparticles of carbon incorporating a non-diffractive 3.23-mm diameter opaque mask with a 1.36-mm central aperture.

The IC-8 could be implanted in conjunction with an aspheric monofocal IOL implantation in the dominant eye, which will improve intermediate and near vision while preserving far vision in the eye with a monofocal optic IOL.^[7]^

The aim of this study was to evaluate the visual acuity and satisfaction after IC-8 implant in patients characterized by previous corneal irregularities and HOAs who cannot undergo refractive surgery due to the poor residual thickness of the cornea or other conditions such as stigmata related to previous radial keratotomy.

##  SURGICAL METHOD

This was a descriptive, retrospective cohort study of nine eyes in six patients affected by corneal irregularities and HOAs who had undergone IC-8 IOL implantation between October 2019 and January 2020 at Careggi Hospital in Florence. All had monolateral implants except for three patients. Patients with significative comorbidities affecting visual acuity such as glaucomatous neuropathy and maculopathy were excluded from the study.

Patients included in the study were subjected to a protocol that provided a baseline and three follow-up visits at day 1, month 1 and month 3, which included Snellen best-corrected visual acuity (BCVA), biomicroscopy, Goldmann applanation tonometry, corneal topography and study of the corneal HOAs (Sirius CSO Florence), fundoscopy (Volk lens) in miosis and after pharmacological dilation, AS-OCT (MS 30, CSO Florence).

The aberrometry (Osiris, CSO Florence) was performed, but the diameter of the opaque mask (1.36 mm central aperture) did not allow the machine's rays to reach a good acquisition. All patients also underwent preoperatively and postoperatively a 30:2 Humphrey visual field and Esterman 120 points visual field.

### Surgical Technique

All 9 IC-8 implants were performed by the same surgeon (FF) after phacoemulsification (clear lens extractions) under topical anesthesia.

We used a dispersive viscoelastic device (OVD) into the anterior chamber and we made a 2.4 mm corneal incision. Then, the surgeon performed a capsulorhexis, hydrodissection, hydrodelineation, and phacoemulsification. All small-aperture IOLs were placed in the capsular bag using the small-aperture IOL injection system with haptic orientation at 12 o'clock and 6 o'clock. IC-8 IOL was centered referring to the Purkinje reflex.

**Figure 1:**
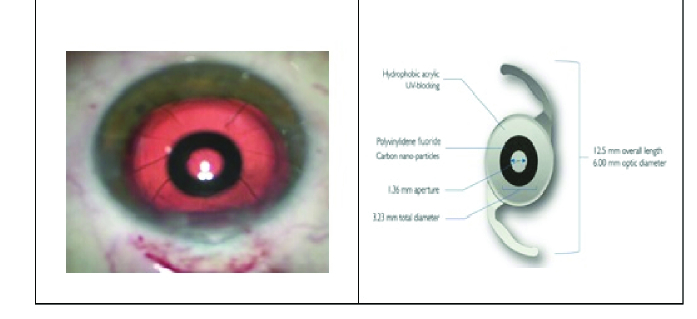
IC-8 IOL placed in the capsular bag, cornea with previous radial keratotomy (left). Small aperture IOL specifications: optic and mask design (right).

Finally, the viscoelastic was removed with irrigation and aspiration and we hydrated the two paracenteses. In this study, no intraoperative or postoperative complications happened [Figure 1].

### Outcomes

Primary outcomes were determined by the improvement of logMAR BCVA and the evaluation of the postoperative visual fields (Humphrey 30:2 and Esterman 120 points). Secondary outcomes were reflected in the increase of visual quality and patients' satisfaction, as revealed from performing the visual function questionnaire 25 (VFQ-25).

Informed consent was obtained from all patients who underwent surgery. The study protocol was approved by the local ethics committee in compliance with the tenets of the Declaration of Helsinki.

We used the VFQ-25 questionnaire to assess self-reported vision-targeted health statuses. Patients were contacted via telephone and answered the questionnaire; the answers were converted into numerical representative values.

**Figure 2:**
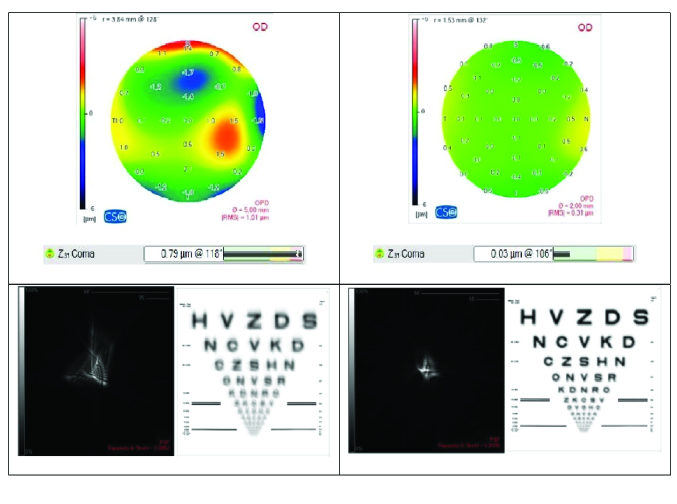
Predictive modifications of Zernicke's summary and visual summary from 5 to 2 mm of corneal diameter (Sirius CSO).

**Figure 3:**
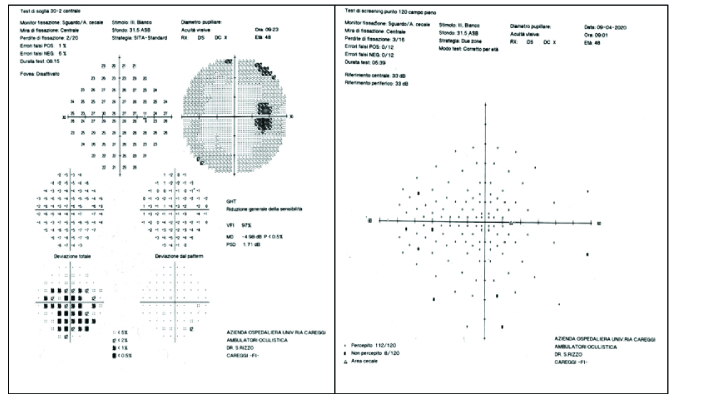
Visual field 30 – 2 (left) and 120 (right): no alterations related to IC8 lens.

**Figure 4:**
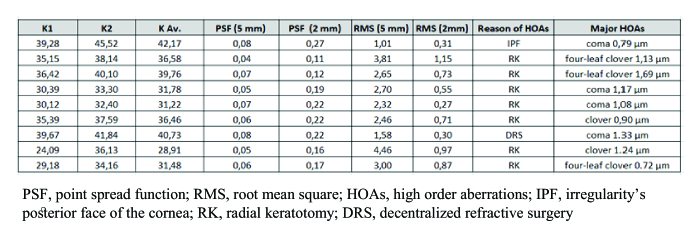
Corneal topography study.

**Figure 5:**
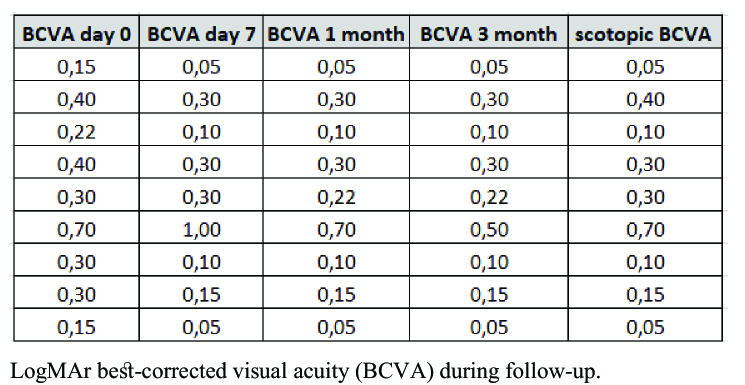
Visual acuity study.

**Figure 6:**
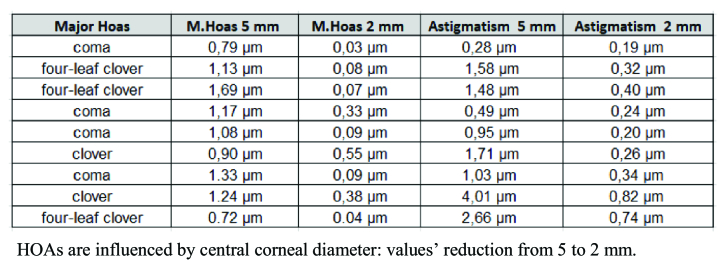
Major HOAs study.

##  RESULTS

The study sample included six patients, two males (33%) and four females (67%), with a mean age of 57 years old, three of which underwent surgery in both eyes.

In total, nine eyes had been subjected to IC-8 implantation: four right eyes (44%) and five left eyes (56%).

Almost the entire sample (78%) presented corneal irregularity due to previous radial keratotomy, one (11%) had a previous decentralized refractive surgery with low residual corneal thickness and one (11%) had a previous pterygiectomy with residual irregularities on the anterior and posterior face of the cornea. In this case, we performed a photo-therapeutic keratectomy (PTK) on the anterior face with an unsuccessful result because of the irregular posterior face.

We evaluated the high order corneal aberration (Sirius CSO Florence): in six eyes (67%) the main HOA was coma (mean value 1.13 µm), in two patients (22%) four-leaf clover (mean value 1.41 µm), and in one patient (11%) clover (0.90 µm) [Table 1].

BCVA increased from a preoperative mean of LogMAR 0.32 ( ± 0.17) to 0.26 ( ± 0.30) on day 7, 0.22 ( ± 0.20) at month 1, and 0.20 ( ± 0.15) at month 3 [Table 2]. The percentage reduction of mean LogMAR BCVA at the end of the follow-up period (month 3) was 25%, compared to the one at the preoperative visit.

Our patients underwent a clear lens extraction and monofocal small aperture lenses were then implanted. This is very important because the prevalence of HOAs is not influenced by cloudy lenses (all HOAs come from corneal shape). The 1.36 mm central aperture and the non-diffractive 3.23 mm diameter opaque mask of the IC-8 recreate the pinhole principle. So, it is possible to “reflect” this effect onto the corneal surface: only the central 2 mm of the cornea becomes fundamental for vision. HOAs are influenced by the central corneal diameter that we focused on: less corneal diameter means little HOAs at topography study. Aberrometric reviews confirmed the reduction of the major HOAs of the patients in our study [Table 3]. Less HOAs meant a better quality of eye vision.

It is important to increase the number of selected patients with irregular cornea and IC-8 implants to ensure the validity of the data obtained. In fact, increasing the selection facilitates greater understanding of establishing the origins of the corneal irregularities and would also assist in determining which specific HOAs would obtain the appropriate results with the implementations IC-8 IOLs.

During this study, there were no instances of intraoperative or postoperative complications.

##  DISCUSSION

We focused our attention on the use of IC-8 IOLs in patients who suffered with cataract, severe corneal irregularities, and HOAs. Generally, when the corneal thickness allowed it, we preferred to correct the corneal irregularities with a customized laser ablation before the cataract surgery was performed. However, in this study, the corneas of patients could not be treated with refractive surgery due to the poor residual thickness of their cornea or presence of other conditions such as posterior corneal astigmatism.

In our study, seven eyes (78%) had previous radial keratotomy (RK), one eye (11%) had a previous decentralized refractive surgery, and one (11%) had a previous pterygectomy with irregularity of the posterior face of the cornea. HOAs cause difficult night vision, glare, halos, blurring, starburst patterns or double vision (diplopia), and cannot be corrected through conventional techniques (i.e., toric IOLs). Furthermore, in these cases, the calculation of IOL power is more challenging since the corneal power is hard to be assessed. In our study, the nine eyes in which the small-aperture IC-8 has been implanted experienced an increase of BCVA, and a subjective vision quality improvement (objective and subjective visual outcomes).

Since the irregularities of the cornea cannot be corrected with customized refractive surgery because of the risk of low residual corneal thicknesses occurring or because of the resulting presence of many radial cuts, we decided to act according to the principles of a pinhole. A small pinhole, which reduce the effective pupil size, leads to a disruption of peripheral rays, while allowing central focused light to reach the retina, and increasing the depth of focus. Ogle et al^[8]^ found that there is an inverse relationship between the pupil size and the depth of focus. Moreover, a relationship could be found between pupil size and HOAs, where small pupils can attenuate the visual effect of corneal aberrations.^[9,10]^

The simulation of the visual quality of the Sirius takes into account the corneal aberrations and demonstrates an aberration index Point Spread Function (PSF describes the shape of an image in an optical system) and the related Strehl Ratio change according to the size of the pupil. In all patients, the PSF value was better at 2 mm (mean value 0.17569) than at 5 mm (mean value 0.06249). So, we decided to implant the IC-8 and take advantage of the small aperture IOL principle to reduce visual aberrations and to improve the PSF [Figure 2].

The pinhole effect has been already exploited by the add-on XtraFocus lens (Morcher), a total black IOL for sulcus implantation, specifically designed for cases with corneal irregularities.^[11,12,13]^ Compared to the XtraFocus lens, which is an add-on lens without optical power, the IC-8 has optical power and can be placed directly in the capsular bag during the cataract surgery, with a 1.36 mm central aperture and a non-diffractive 3.23 mm diameter opaque mask that allows the possibility of studying the retina, and also performing retinal surgery.

After the implantation of the IC-8 IOL, we achieved a decrease of LogMAR BCVA from a preoperative mean of 0.35 ( ± 0.18) to 0.25 ( ± 0.22) at month 3. Subjective improvement of self-reported vision-targeted health status was observed as patients reported their satisfaction while answering the VFQ-25 questionnaire. Especially, after receiving bilateral implants, patients reported that they felt more confident in daily life.

The preoperative visual impairment that patients experienced was mainly related to the presence of HOAs and on a smaller scale due to the presence of cataract.

Our findings confirm data reported in prior literature: Shajari^[3]^ reported in 17 eyes an improvement of corrected distance visual acuity (CDVA) from the preoperative value of 0.37 to 0.19 LogMAR at month 3 after the IC-8 lens implantation in eyes with severe corneal irregularities. Grabner et al^[14]^ registered a similar result. Schultz and Dick reported the use of the IC-8 lens in a case of a patient with irregular cornea because of trauma: after surgery they experienced an improvement in visual acuity across all distances.^[15]^ Agarwal^[16]^ implanted IC-8 IOL in patients who had previous refractive surgery and HOAs: she reported that the small-aperture IOL reduced the effects of HOAs on visual acuity and provides continuous depth of focus.

Examination of the posterior segment was always possible, although in non-mydriatic conditions, during the follow-up visits. The mask only extends to 3.23 mm in total, so that peripheral IOL is clear and doesn't preclude visualization of the peripheral retina with Volk lens. These findings corroborate Srinivasan's^[17]^ data from 15 patients: optical coherence tomography, posterior segment investigations including fundus photography, and perimetry could be safely performed in eyes with IC8 IOLs. Moreover, Srinivasan et al did not report any issues according to the intraoperative view and the surgeon could perform all vitrectomy procedures, without distortions and disc halos on the retina, as is common with diffractive and refractive multifocal lenses.^[18]^

Despite the presence of the opaque mask, measuring 3.23 mm, the field of vision was maintained. We performed a visual field (VF) Humphrey 30:2 and a full-field 120-point screening test, where, even with the presence of the opaque mask, in the most extreme periphery of the visual field we did not detect any shrinkage or defect. We achieved the visual fields even in pharmacological mydriasis conditions to assess whether the visual impairment due to the mask would have been greater in a situation mimicking low light conditions. Both conditions of the VF were performed at month 3 of follow-up to ensure that the patients were adapting to the presence of the IC-8 IOL. In our study, we registered a good subjective adaptation, without any resultant shrinkage in visual field, in both bilateral (50% of the cases) and monolateral cases [Figure 3].

No adverse events occurred during the follow-up. Only one patient developed significant posterior capsule opacification (PCO) that required YAG laser capsulotomy only in the 1.36 mm central aperture. Previous works have reported a small PCO rate after IC-8 implantation (6.8% in Ang's work,^[19]^ 7% in Shajari's study,^[20]^ and 12% in Hooshmand's study,^[21]^ which were treated with the YAG laser without further complications.

In summary, the result of our work encourages the use of the IC-8 lens to improve visual acuity in patients with irregular corneas and HOAs that cannot be treated with customized refractive surgery. Patients feel a subjective improvement in their quality of vision and more confident in daily life. The IC-8 lens does not interfere with visualization of fundus and no impairment of the visual field are detected by patients.

##  Financial Support and Sponsorship

None.

## Conflicts of Interest

There are no conflicts of interest.
